# Platelet-T cell aggregates in lung cancer patients: Implications for thrombosis

**DOI:** 10.1371/journal.pone.0236966

**Published:** 2020-08-10

**Authors:** Claire K. Meikle, Adam J. Meisler, Cara M. Bird, Joseph A. Jeffries, Nabila Azeem, Priyanka Garg, Erin L. Crawford, Clare A. Kelly, Tess Z. Gao, Leah M. Wuescher, James C. Willey, Randall G. Worth

**Affiliations:** 1 Department of Medical Microbiology & Immunology, University of Toledo College of Medicine and Life Sciences, Toledo, OH, United States of America; 2 Department of Medicine, University of Toledo College of Medicine and Life Sciences, Toledo, OH, United States of America; Institut d'Investigacions Biomediques de Barcelona, SPAIN

## Abstract

Platelet-leukocyte aggregates (PLAs) are associated with increased thrombosis risk. The influence of PLA formation is especially important for cancer patients, since thrombosis accounts for approximately 10% of cancer-associated deaths. Our objective was to characterize and quantify PLAs in whole blood samples from lung cancer patients compared to healthy volunteers with the intent to analyze PLA formation in the context of lung cancer-associated thrombosis. Consenting lung cancer patients (57) and healthy volunteers (56) were enrolled at the Dana Cancer Center at the University of Toledo Health Science Campus. Peripheral blood samples were analyzed by flow cytometry. Patient medical history was reviewed through electronic medical records. Most importantly, we found lung cancer patients to have higher percentages of platelet-T cell aggregates (PTCAs) than healthy volunteers among both CD4+ T lymphocyte and CD8+ T lymphocyte populations. Our findings demonstrate that characterization of PTCAs may have clinical utility in differentiating lung cancer patients from healthy volunteers and stratifying lung cancer patients by history of thrombosis.

## Introduction

Lung cancer is the most common cause of cancer deaths in the United States [1]. Lung cancer patients are 100 times more likely to develop thrombosis than the general population therefore making lung cancer-associated thrombosis a significant health problem [2]. Lung cancer increases risk of venous thromboembolism (VTE) four- to seven-fold [3] and also increases risk of arterial thrombotic events (ATE) [4]. Importantly, thrombosis is a leading cause of death in cancer patients, second only to progression of cancer [5] and patients with cancer-associated VTE have a two- to six-fold higher risk of death compared to patients with thrombosis that do not have cancer [6, 7]. Cancer-associated thrombosis is linked to cisplatin [8], thrombocytosis [9, 10] and cancer patients on chemotherapy with a platelet count > 350,000/μL had a threefold increase in frequency of VTE [11]. ATE more than doubles the risk of death in cancer patients and quadruples risk of death specifically in lung cancer patients [12, 13]. Most importantly, a recent report found that ATE predicted a diagnosis of cancer, including lung cancer, within 150 days of the thrombotic event [14].

Platelets are known to influence cancer progression and the tumor microenvironment facilitates platelet activation [15]. Tumor cells can activate platelets and thereby promote thrombosis by producing tissue factor and tissue factor expressing microparticles [16, 17], matrix metalloproteinases [18], and thrombin (with other prothrombotic stimuli) [19, 20]. Platelet activation is characterized by shape change, degranulation, and upregulation of platelet surface receptor expression, facilitating platelet binding to endothelium, fibrinogen, leukocytes, and other platelets. Importantly, P-selectin is expressed on the surface of activated platelets and mediates platelet adhesion to leukocytes and tumor cells [21–23]. Soluble P-selectin has been shown to be increased in patients with VTE, and has clinical utility as a predictive marker [24].

Platelet-leukocyte interactions are used to assess cancer treatment and thrombosis risk. Elevated platelet-lymphocyte ratio may be used as a prognostic, staging, and follow up tool [25–32] and is a predictor of VTE risk in cancer patients [33]. Patients with myeloproliferative disorders have high numbers of platelet-leukocyte aggregates (PLAs) compared to healthy volunteers, and the percentage of PLAs was correlated with higher P-selectin expression on platelets and history of previous thrombosis [34]. Moreover, PLA formation is increased in patients with acute myocardial infarction [35]. These results suggest that PLAs and platelet activation may contribute to thrombosis in select patient populations. P-selectin facilitates progression of atherosclerotic lesions, stimulating and mediating binding of platelets to monocytes and macrophages [36]. However, it remains unknown if PLA formation may influence thrombosis risk in cancer patients. Therefore, the aim of this study is to examine the relationship between cancer-associated thrombosis, PLA formation, and P-selectin expression in patients with lung cancer.

## Materials and methods

### Study design and enrollment

Lung cancer patients were approached during a visit to the University of Toledo Dana Cancer Center between July 2016 and December 2019. At the time of consent, the cancer status of the patient was unknown. Fifty-seven individuals with lung cancer and56 healthy volunteers were consented and enrolled as. After consent, patients and healthy volunteers completed a brief questionnaire to determine basic demographics, smoking history, previous thrombotic events, and current medications. If healthy volunteers indicated they have active cancer they were excluded from the study. Subsequently, patient medical records were assessed for cancer type, stage, previous thrombotic event, and current and previous medications (chemotherapies, anti-coagulants/anti-platelets). Peripheral blood samples were collected from all study participants through antecubital vein directly into 10% ACD vacutainers (16 x 100mm, BD Biosciences, San Jose, CA).

### Reagents

All monoclonal antibodies were purchased from Biolegend (San Diego, CA, USA) (S1 Table). Platelets were identified by flow cytometry using allophycocyanin (APC) conjugated anti-CD42b (GPIbα) (clone HIP1). Platelet P-selectin expression was detected using PE-Cy5-conjugated anti-CD62P (P-selectin) (clone AK4). Leukocyte populations were defined using the following monoclonal antibodies: leukocytes–phycoerythrin (PE) conjugated anti-CD45 (clone HI30), T cells–FITC-conjugated anti-CD3 (UCHT1), neutrophils–PerCP/Cy5.5-conjugated anti-CD66B (clone G10F5), monocytes–PE-conjugated anti-CD14 (clone63D3), natural killer cells–PerCP/Cy5.5-conjugated anti-CD56 (clone HCD56), CD4 T cells–FITC-conjugated anti-CD4 (clone RPA-T4), CD8 T cells–FITC-conjugated anti-CD8 (clone RPA-T8), and B cells–FITC-conjugated anti-CD19 (clone HIB19).

### Whole blood staining

Whole blood was labeled with fluorescently conjugated isotype control antibodies or anti-CD42b and -CD62P, and a combination of anti-CD45, -CD25, -CD14, -CD66B, -CD56, -CD3, -CD4, -CD8, or -CD19 for 30 minutes on ice in the dark. Cells were washed with calcium and magnesium-free PBS, fixed for at least 1 hour in 2% paraformaldehyde, and then washed and resuspended in PBS. Flow cytometry was performed using a FACSCalibur flow cytometer (BD Biosciences, San Jose, CA) and analyzed using FlowJo software (Tree Star, Ashland, OR; Version 7.6.5).

### Flow cytometry gating

Populations were identified by marker expression (compared to isotype control labeled samples), then further gated by forward scatter vs side scatter (S1 Fig). Monocytes were gated first on CD3-negative events, then on CD14-positive events, then were gated by forward scatter vs side scatter. PLAs were identified based on the number of events expressing CD42b out of the total parent population, and CD62P expression was further quantified in the PLA parent population. Mean fluorescence intensity (MFI) of CD42b for the total platelet population and CD62P for the activated platelet population was determined. Aggregate fluorescence intensity was calculated by dividing CD42b fluorescence for aggregates by the average MFI of an average healthy platelet (239 units, Table 1).

**Table 1:** Clinical characteristics of enrolled individuals.

	Lung Cancer Patients Mean (range)	Healthy Volunteers Mean (range)	*P*
Number of individuals	57	56	-
Sex (M:F)	31:26	26:30	0.3976
Age	66.72 (44–84)	47.52 (22–92)	0.0001
Pack-years	43.12 (0–165)	10.42 (0–72)	0.0001
History of thrombosis	18	2	0.0001
Type of cancer			
Adenocarcinoma	35	-	
Squamous cell	8	-	
Other	14	-	
% CD4+ cells	0.57% (0.0008% - 5.38%)	0.47% (0.0008% - 4.36%)	0.3238
% CD8+ cells	0.36% (0.0002% - 2.09%)	0.54% (0.03% - 7.71%)	0.2040
% Platelets	14.2% (2.26% - 47.97%)	15.4% (0.20% - 51.68%)	0.6141
% Activated Platelets	6.85% (0.30% - 20.38%)	3.81% (0.26% - 54.08%)	0.0565
CD42b expression (MFI)	200.9 (78.4–1078)	239.0 (84.3–1029)	0.6513
CD62P expression (MFI)	29.12 (3.76–508)	11.69 (2.37–314)	0.0005

### Statistics

Data were analyzed using chi-square analysis for categorical data, nonparametric Mann Whitney test for comparisons between two groups, nonparametric one-way Kruskal-Wallis test with Dunn’s multiple comparison post-hoc test for comparisons between three groups, two-way ANOVA with Bonferroni post-test for grouped comparisons, Wilcoxon matched-pairs signed rank test for paired analyses. Linear regression receiver operating characteristic (ROC) curves were generated as a function of sensitivity vs 1-specificity. Area under the curve (AUC) was calculated. Likelihood ratio (LR) was calculated as sensitivity divided by 1-specificity. The percentage of lung cancer patients that fell outside of the healthy confidence interval was determined for each parameter analyzed. All data were analyzed in Prism 5.03 software (GraphPad) and p ≤ 0.05 was considered statistically significant.

### Study approval

All participants were consented into the University of Toledo Biomedical IRB-approved study and consistent with the Declaration of Helsinki. Written informed consent was received from participants prior to inclusion in the study.

## Results

A total of 57 lung cancer patients and 56 healthy volunteers were enrolled (Table 1), comprising roughly equal numbers of male and female subjects. The lung cancer patient population was significantly older with more pack-years than the healthy volunteer population, but age and smoking history are not correlated with the parameters assessed in this study (S2 and S3 Figs). Cancer patients also are more likely to have experienced a past thrombotic event (either VTE or ATE) than healthy volunteers. Non-small cell lung cancer (NSCLC), including adenocarcinoma and squamous cell carcinoma, was the most common type of cancer among lung cancer patients (n = 43), and the most common type of NSCLC was adenocarcinoma (n = 35, Table 1), consistent with previously-reported population studies [37].

Baseline blood cell percentages were obtained via flow cytometry and showed that lung cancer patients and healthy volunteers did not differ in the baseline percentage of CD4+ cells, CD8+ cells, nor platelets in whole blood (Table 1). No difference in CD42b (GPIbα) expression was detected between healthy volunteers or lung cancer patients. However, lung cancer patients tended to have a higher percentage of activated platelets than healthy volunteers (Table 1). Correspondingly, lung cancer patients express significantly higher levels of CD62P (P-selectin) than healthy volunteers (Table 1).

**Fig 1:**
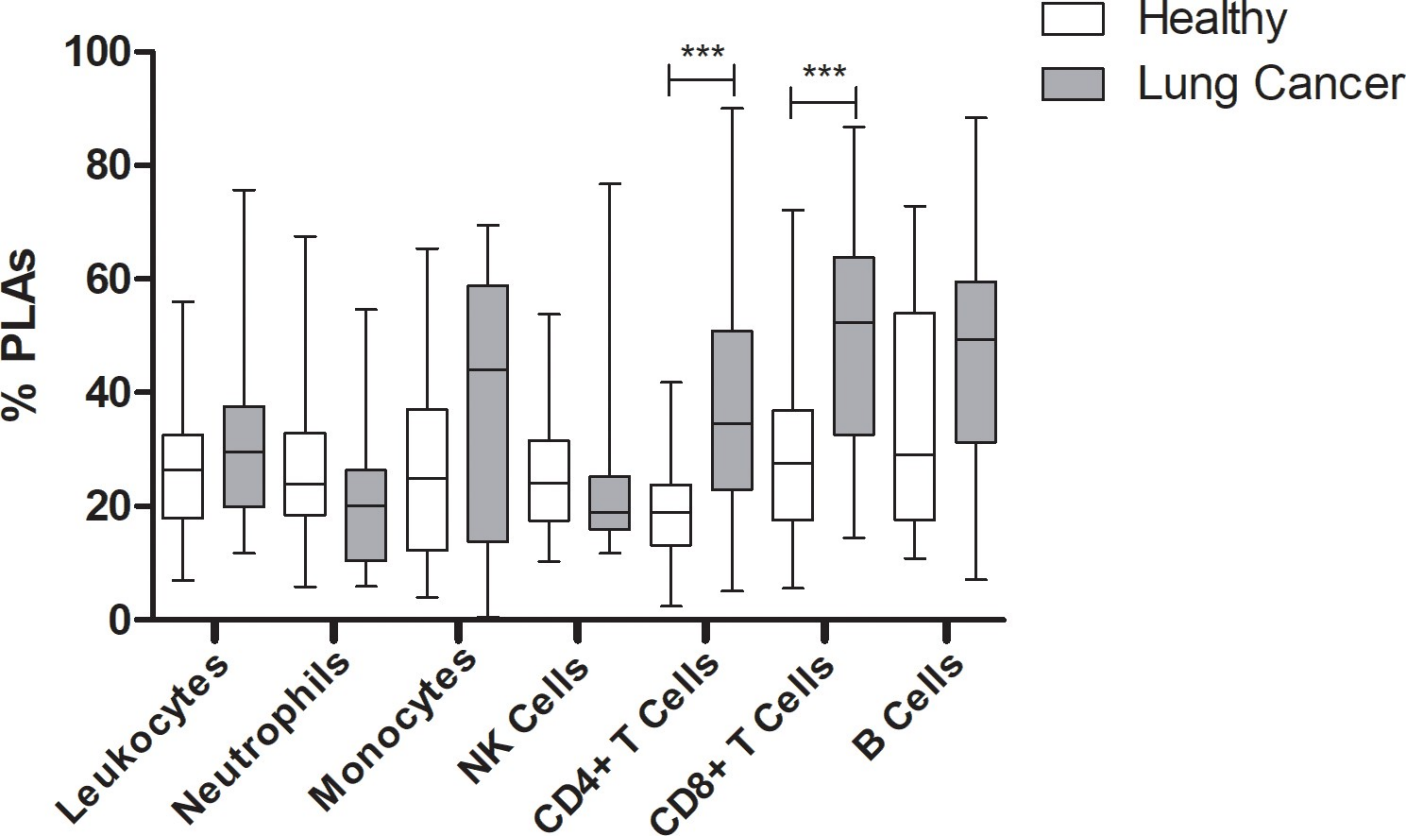
Platelet-leukocyte aggregates in lung cancer patients compared to healthy volunteers. (A) Whole blood from healthy volunteers (white) or lung cancer patients (gray) was labeled with markers for leukocytes (anti-CD45), neutrophils (anti-CD66B), monocytes (anti-CD3 negative, anti-CD14+), natural killer cells (anti-CD56), CD4+ T cells (anti-CD4), CD8+ T cells (anti-CD8), or B cells (anti-CD19). Samples were co-labeled with APC-conjugated anti-CD42b. Mann Whitney nonparametric test. *** p ≤ 0.001.

PLAs have been shown to increase the risk of thrombosis [34, 35], and lung cancer patients are known to be at increased risk of thrombosis [38–42]. Therefore, we hypothesized that PLAs may be increased in this population. Whole blood samples were gated based on surface receptor expression for each leukocyte subset and were further gated based on forward scatter vs side scatter (S1 Fig) to better define the population. Since PLAs have not been assessed in cancer patients, we assessed several leukocyte populations for interaction with platelets: total leukocytes, neutrophils, monocytes, natural killer cells, T cells (CD4+ and CD8+), and B cells. Surprisingly, lung cancer patients did not show increased platelet aggregation with neutrophils nor monocytes, as has been reported in other prothrombotic conditions [43] (Fig 1A). However, platelet-CD4+ and platelet-CD8+ T cell aggregates were each increased in lung cancer patients compared to healthy volunteers (Fig 1A). Therefore, interactions between platelets and CD4+ and CD8+ populations were further investigated.

**Fig 2:**
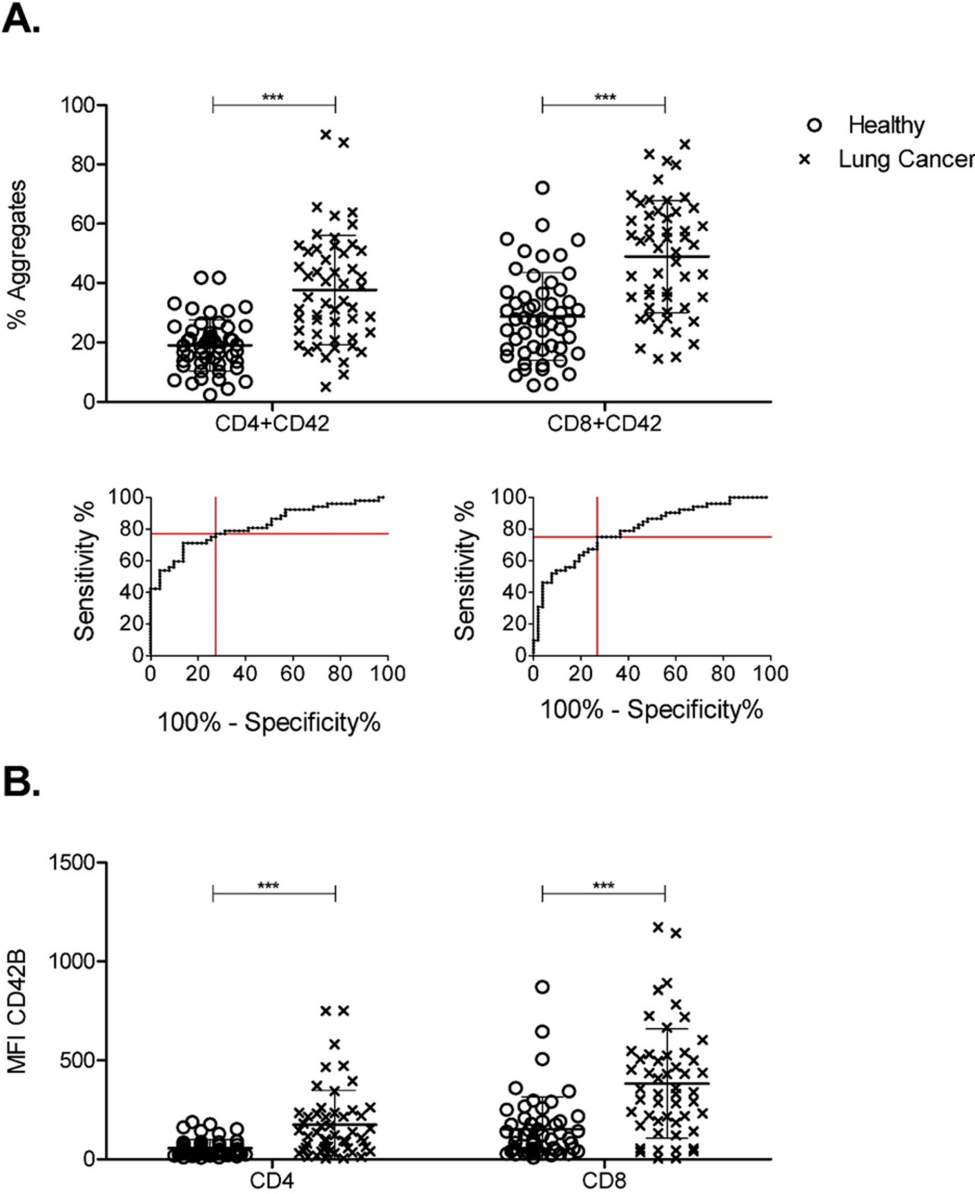
Frequency and size of platelet-T cell aggregates in lung cancer patients compared to healthy volunteers. Whole blood from healthy volunteers or lung cancer patients was labeled with markers for CD4+ T cells (anti-CD4) or CD8+ T cells (anti-CD8) and were co-labeled for platelets (anti-CD42b). Data was collected by flow cytometry. Populations were gated based on CD4+ and CD8+. Percent of T cells with a platelet attached (A) and MFI of platelets (B) were calculated using FlowJo software. ROC curves were generated for CD4+ and CD8+ PTCAs for lung cancer patients compared to healthy volunteers. Optimal sensitivity and (1-specificity) were determined using GraphPad Prism and are plotted as red lines. Mann Whitney nonparametric test. Error bars represent mean ± SD. N = 44–52. ** p ≤ 0.01, *** p ≤ 0.001.

Both CD4+ and CD8+ PTCAs were detected more frequently in lung cancer patients than in healthy volunteers (Fig 2A). To ascertain clinical significance, receiver operating characteristic (ROC) curves were generated for each parameter in Fig 2 (S4 Fig). ROC analysis yielded sensitivity, specificity, and likelihood ratio for optimal detection limits for each parameter (Table 2). Every parameter except for percent of activated platelets was found to be a significant predictor of lung cancer (Table 2). Percent of CD4+ and CD8+ PTCAs showed the strongest predictive value. Interestingly, in addition to being more frequent, lung cancer patient PTCAs showed a higher MFI of CD42b (both CD4+ and CD8+) than those from healthy volunteers (Fig 2B) which may suggest more platelets per T cell. However, this needs further investigation.

**Table 2:** Clinical significance of PTCAs as a predictor of lung cancer.

Parameter	Detection Limit	AUC	Sens. (%)	Spec. (%)	95% CI Healthy	95% CI Lung Cancer	% Lung Cancer greater than Healthy CI
% CD4+CD42b	>22.43%	0.80***	76.92	72.55	(17.38–22.62)	(32.58–42.84)	76.9
% CD8+CD42b	>35.24%	0.79***	75.00	73.08	(25.55–34.45)	(43.70–54.24)	75
% CD42b+CD62P	>2.265%	0.61	62.26	48.00	(2.62–4.99)	(4.58–8.74)	38.5
% CD4+CD42b+CD62P	>29.09%	0.67**	67.39	59.09	(21.47–35.46)	(36.49–53.81)	56.5
% CD8+CD42b+CD62P	>47.57%	0.68**	63.83	60.87	(27.78–45.03)	(46.60–62.18)	63.8
MFI CD42b of CD4+	>51.05	0.77***	71.70	69.23	(42.93–67.42)	(126.3–223.6)	65.4
MFI CD42b of CD8+	>191.5	0.78***	75.00	75.00	(106.5–196.9)	(304.6–459.8)	71.2
MFI CD62P of CD42b	>9.370	0.69***	66.07	68.33	(8.38–15.00)	(10.69–47.55)	36.5
MFI CD62P of CD4+CD42b	>55.15	0.67**	62.22	69.57	(39.59–89.74)	(71.13–165.3)	33.3
MFI CD62P of CD8+CD42b	>79.95	0.686**	63.04	64.44	(55.27–125.4)	(108.2–211.2)	45.7

ROC curves were generated and area under the curve (AUC) was calculated with respective p-values. AUC curves were used to identify optimum sensitivity (Sens) and specificity (Spec). 95% confidence intervals for lung cancer patients and healthy volunteers. Confidence intervals calculated using GraphPad Prism. The percentage of lung cancer patients that fell outside of the healthy confidence interval was determined for each parameter.

** p ≤ 0.01

*** p ≤ 0.001.

**Fig 3:**
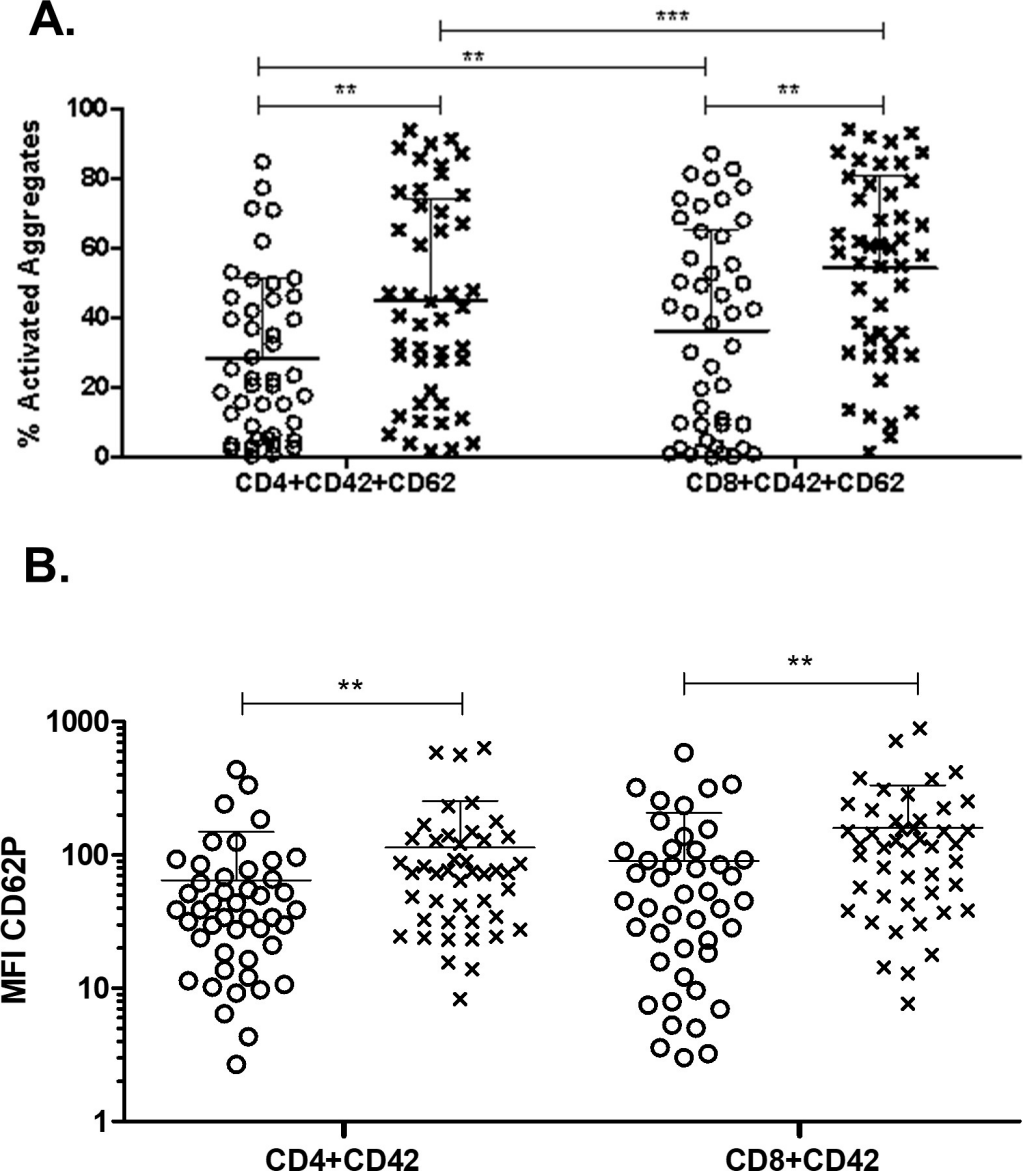
Platelet activation within platelet-T cell aggregates in lung cancer patients compared to healthy volunteers. Whole blood from healthy volunteers or lung cancer patients was labeled with markers for CD4+ T cells (anti-CD4) or CD8+ T cells (anti-CD8) and were co-labeled for platelets (anti-CD42b) and activated platelets (anti-CD62P, P-selectin). Data was collected by flow cytometry. Populations were gated based on CD4+ and CD8+. PTCAs were identified as in Fig 2. Populations were further gated based on PTCAs and the percent of activated platelets (A) and MFI of activated platelets (B) within PTCAs were calculated. Mann Whitney nonparametric test. Error bars represent mean ± SD. N = 44–52. ** p ≤ 0.01, *** p ≤ 0.001.

Platelet activation within PTCAs was analyzed by quantifying the percent of PTCAs positive for P-selectin expression. Lung cancer patients had more activated platelets in both CD4+ and CD8+ PTCAs (Fig 3A). Similarly, a higher percentage of platelets in both CD4+ PTCAs and CD8+ PTCAs were activated compared to free platelets in both healthy volunteers and lung cancer patients (not shown). These results indicate that platelets attached more frequently to T cells in lung cancer patients than healthy volunteers, and that a higher percentage of those platelets were activated in lung cancer patients than in healthy volunteers. However, it should be stated that considering the large variability, platelet activation within PCTAs most likely is not a useful clinical finding.

The magnitude of platelet activation in PTCAs was characterized by MFI of P-selectin. P-selectin expression was increased in free platelets from lung cancer patients compared to healthy volunteers (Table 1). Moreover, P-selectin expression was higher in lung cancer patient CD4+ and CD8+ PTCAs compared to those from healthy volunteers (Fig 3B). We then investigated differences between the PTCAs (CD4+ vs. CD8+). These results suggest that platelets in lung cancer PTCAs are more activated than those from healthy volunteers.

Many lung cancer patients enrolled in our study were prescribed antiplatelet or anticoagulant medications (Table 3). In order to determine whether these medications affected PTCA formation, patients were subdivided into those who had taken antiplatelet or anticoagulant medication (e.g., aspirin, clopidogrel, cilostazol, warfarin, NSAIDs, acetaminophen; Meds) and those that had not (No Meds). We detected no differences in PTCA characteristics between patients currently on medications compared to those not on medication (S5 Fig).

**Table 3:** Antiplatelet and anticoagulant medications.

	None	ATE	VTE	Total
Antiplatelet	1	3	2	6
Cilostazol	0	0	1	1
Clopidogrel	1	3	1	5
Anticoagulant	1	3	2	6
Apixaban	1	1	1	3
Warfarin	0	2	1	3
COX Inhibitor	41	11	6	58
Aspirin	15	8	2	25
NSAID	9	1	1	11
Acetaminophen	6	2	2	10
None	11	0	1	12

## Discussion

Frequency of PTCAs were increased in lung cancer patients compared to healthy volunteers, both in number and size of aggregates. Indeed, characterization of PTCAs demonstrates clinically significant ability to differentiate lung cancer patients from healthy volunteers. For example, analysis of blood samples showing PTCA frequency greater than 23% may suggest the patient may have lung cancer. PTCAs have not previously been studied in lung cancer patients, but research on CD45+ leukocytes demonstrated a link between PLAs and previous thrombosis in myeloproliferative disorders [34]. Tumor cells are able to induce platelet aggregation [18, 44–46], and this may be a contributing factor toward increased PTCAs in lung cancer patients. Platelets attach to T cells in the context of inflammation [47–49] and T cells have been shown to bind to platelets, thereby preventing hemorrhagic transformation in ischemic stroke [50]. Interestingly, more platelets attached to CD8+ cells than to CD4+ cells, and those platelets expressed P-selectin. Platelets within aggregates expressed more P-selectin than free platelets, indicating that platelet activation may play a key role in mediating PTCA formation. The specific mechanism underlying platelet-T cell attachment observed in this study remains unknown, and further research to elucidate the underlying mechanism is warranted.

Platelets can modulate and release soluble factors to influence tumor cells and immune cells. Platelets have been shown to suppress T cell anti-tumor activity by activating latent free TGF-β [51]. Further, tumor cells are able to hijack this process to facilitate oncogenesis [52]. Our results demonstrate direct platelet-T cell attachment in the context of cancer, and it is possible that direct binding may also influence T cell function in cancer patients. While our results have implications for the role of PTCAs in immune function, further studies are needed to establish an immunomodulatory function of PTCAs.

Cancer patients commonly require antiplatelet or anticoagulant medications for thrombosis treatment or prophylaxis, but long-term use of antiplatelet agents increases risk of bleeding and gastrointestinal side effects and is not recommended for most patients [53, 54]. However, even when cancer patients are treated with an anticoagulant, they remain at high risk for recurrent thrombosis [55, 56]. Increased soluble P-selectin in serum is a risk factor for recurrent thrombosis [57]. Notably, whether lung cancer patients were taking any type of antiplatelet or anticoagulant drug did not influence PTCA formation nor P-selectin expression within PTCAs (not shown), indicating that PTCA formation may provide clinically useful insight with respect to cancer and thrombosis risk, including recurrent thrombosis. It should however be noted that cancer-associated thrombosis is a complex and multifactorial process that could be influenced by tumor cell and platelet microparticles, tissue factor, other coagulation factors, and endothelial dysfunction which were not accounted for in this study.

This single-center cohort with relatively small sample size should be understood as a limitation of this study. Although these results show correlations in lung cancer patients, it is unknown if similar observations will be seen in patients with other cancer types. A larger scale multi-center study should be undertaken to account for external validity and to have a larger cohort so that comparisons between different lung cancer histology’s can be evaluated.

More research is needed to evaluate the predictive value of PTCA formation in assessing risk of arterial and venous thrombosis. Further research is also needed to determine the role of PTCA formation in development of thrombosis and cancer progression, as well as studies to understand the mechanism underlying PTCA formation. This may lead to identification of clinical intervention that could specifically target platelets’ dual role in thrombosis and cancer progression without the global side effects that accompany current antiplatelet treatments.

These findings indicate that interactions between platelets and T cells in cancer patients may contribute to a procoagulant phenotype, possibly explaining why use of anticoagulants or antiplatelet medications are largely ineffective in controlling recurrent thrombosis. Future studies will explore the mechanism underlying platelet-T cell aggregation and clinical applications.

## Supporting information

S1 Table Antibodies and cell populations. (DOCX)

S1 Fig Representative leukocyte gating strategy. CD4+ T cells used as example, other leukocyte subpopulations analyzed similarly. Sample incubated with anti-CD4, anti-CD42b, and anti-CD62P shown in black, respective triple-labeled isotype shown in gray. Histogram gating was used to identify the leukocyte subpopulation surface marker (A). Due to autofluorescence in the neutrophil population, Forward vs Side Scatter was used to refine the subpopulation (B). To identify PLAs, leukocyte subpopulations were gated on CD42b (C). PLAs were assessed for platelet activation by gating on CD62P (D).
(DOCX)

S2 Fig PTCA formation as a variable of age. Whole blood from healthy volunteers (○) or lung cancer patients (x) was labeled with markers for CD4+ T cells (anti-CD4) or CD8+ T cells (anti-CD8) and were co-labeled for platelets (anti-CD42b) and activated platelets (anti-CD62P, P-selectin). Data was collected by flow cytometry. Populations were gated based on CD4+ and CD8+. Percent of CD4+ cells with a platelet attached (A) and percent of CD8+ cells with a platelet attached (B) were calculated using FlowJo software. Populations were further gated based on PTCAs and the percent of activated platelets within CD4+ PTCAs (C) and CD8+ PTCAs (D) were calculated. Linear regression. N = 44–52.
(DOCX)

S3 Fig PTCA formation as a variable of smoking history. Pack-years were calculated as number of packs of cigarettes smoked per day multiplied by number of years participants smoked. Whole blood from healthy volunteers (○) or lung cancer patients (x) was labeled with markers for CD4+ T cells (anti-CD4) or CD8+ T cells (anti-CD8) and were co-labeled for platelets (anti-CD42b) and activated platelets (anti-CD62P, P-selectin). Data was collected by flow cytometry. Populations were gated based on CD4+ and CD8+. Percent of CD4+ cells with a platelet attached (A) and percent of CD8+ cells with a platelet attached (B) were calculated using FlowJo software. Populations were further gated based on PTCAs and the percent of activated platelets within CD4+ PTCAs (C) and CD8+ PTCAs (D) were calculated. Linear regression. N = 44–52.
(DOCX)

S4 Fig ROC curves for lung cancer patients compared to healthy volunteers. Whole blood from lung cancer patients and healthy volunteers was prepared as in Fig 6. Populations were gated based on CD4+ and CD8+. Percent of T cells with a platelet attached (A, B) and MFI of platelets (F, G) were calculated using FlowJo software. Populations were further gated based on PTCAs and the percent of free platelets (C) and MFI of free platelets (H) were determined. Percent of activated platelets (D, E) and MFI of activated platelets (I, J) within PTCAs were calculated. Optimal sensitivity and (1-specificity) were determined using GraphPad Prism and are plotted as gray lines. N = 44–52.
(DOCX)

S5 Fig Platelet-T cell aggregates in lung cancer patients by medication. Whole blood from lung cancer patients taking antiplatelet or anticoagulant medications (Meds) and those not taking any of these medications (No Meds) was prepared as in Fig 6. Populations were gated based on CD4+ and CD8+. Percent of T cells with a platelet attached (A) and MFI of platelets (C) were calculated using FlowJo software. Populations were further gated based on PTCAs and the percent of activated platelets (B) and MFI of activated platelets (D) within PTCAs were calculated. Asterisks above columns indicate a significant difference from the corresponding “CD42+CD62” (B) or “CD42” (D) free platelet population. Two-way ANOVA with Bonferroni post-test and Wilcoxon matched-pairs signed rank test. Error bars represent mean ± SD. N = 14–37. * p ≤ 0.05, ** p ≤ 0.01, *** p ≤ 0.001.
(DOCX)
