# Supplementary material for: Platelet-T cell aggregates in lung cancer patients: Implications for thrombosis
Source: PLoS One. 2020 Aug 10;15(8):e0236966. doi: 10.1371/journal.pone.0236966 (PMC7416940; doi:10.1371/journal.pone.0236966)
Supplement: S1 Table — (DOCX) [file pone.0236966.s001.docx]

**S1 Table. Antibodies and Cell Populations.**

| **Antibody** | **Clone** | | **Fluorophore** | **Stock Concentration (mg/ml)** | |
| --- | --- | --- | --- | --- | --- |
| CD3 | UCHT1 | | FITC | 0.2 | |
| CD4 | RPA-T4 | | FITC | 0.4 | |
| CD8 | RPA-T8 | | FITC | 0.2 | |
| CD14 | 63D3 | | PE | 0.2 | |
| CD19 | HIB19 | | FITC | 0.4 | |
| CD42b | HIP1 | | APC | 0.1 | |
| CD45 | HI30 | | PE | 0.2 | |
| CD56 | HCD56 | | PerCP/Cy5.5 | 0.1 | |
| CD62P | AK4 | | PE-Cy5 | 0.1 | |
| CD66B | G10F5 | | PerCP/Cy5.5 | 0.5 | |
|  |  | |  |  | |
| **Leukocyte Aggregates** | | **Lineage Markers** | | |  |
| T helper cells | | CD4+CD42b+ | | |  |
| Cytotoxic T cells | | CD8+CD42b+ | | |  |
| Neutrophils | | CD66B+CD45+CD42b+ | | |  |
| NK Cells | | CD56+CD45+CD42b+ | | |  |
| B cells | | CD19+CD42b+ | | |  |
| Monocytes | | CD14+CD45+CD3-CD42b+ | | |  |
| Platelets | | CD42b+ | | |  |
| Activated Platelets | | CD42b+CD62P+ | | |  |
| Activated PTCA | | CD4/8+CD42b+CD62P+ | | |  |
