# Supplementary material for: Platelet-T cell aggregates in lung cancer patients: Implications for thrombosis
Source: PLoS One. 2020 Aug 10;15(8):e0236966. doi: 10.1371/journal.pone.0236966 (PMC7416940; doi:10.1371/journal.pone.0236966)
Supplement: S1 Fig — CD4+ T cells used as example, other leukocyte subpopulations analyzed similarly. Sample incubated with anti-CD4, anti-CD42b, and anti-CD62P shown in black, respective triple-labeled isotype shown in gray. Histogram gating was used to identify the leukocyte subpopulation surface marker (A). Due to autofluorescence in the neutrophil population, Forward vs Side Scatter was used to refine the subpopulation (B). To identify PLAs, leukocyte subpopulations were gated on CD42b (C). PLAs were assessed for platelet activation by gating on CD62P (D). (DOCX) [file pone.0236966.s002.docx]

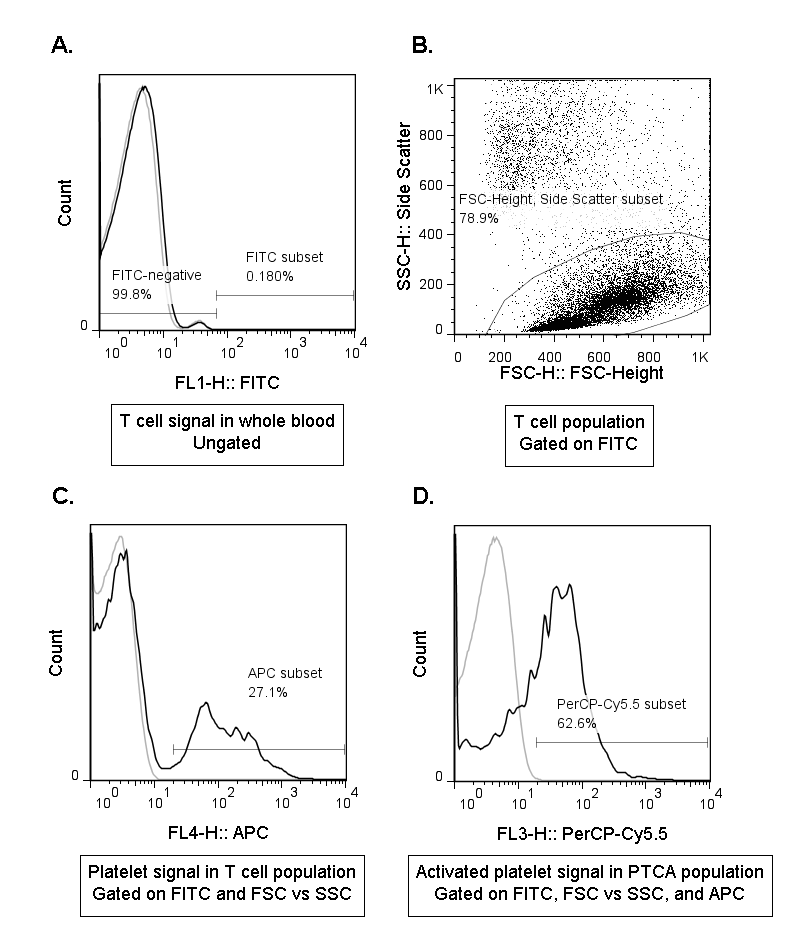


**Figure S1. Representative leukocyte gating strategy.** CD4+ T cells used as example, other leukocyte subpopulations analyzed similarly. Sample incubated with anti-CD4, anti-CD42b, and anti-CD62P shown in black, respective triple-labeled isotype shown in gray. Histogram gating was used to identify the leukocyte subpopulation surface marker (A). Due to autofluorescence in the neutrophil population, Forward vs Side Scatter was used to refine the subpopulation (B). To identify PLAs, leukocyte subpopulations were gated on CD42b (C). PLAs were assessed for platelet activation by gating on CD62P (D).
