# Supplementary material for: Platelet-T cell aggregates in lung cancer patients: Implications for thrombosis
Source: PLoS One. 2020 Aug 10;15(8):e0236966. doi: 10.1371/journal.pone.0236966 (PMC7416940; doi:10.1371/journal.pone.0236966)
Supplement: S3 Fig — Pack-years were calculated as number of packs of cigarettes smoked per day multiplied by number of years participants smoked. Whole blood from healthy volunteers (○) or lung cancer patients (x) was labeled with markers for CD4+ T cells (anti-CD4) or CD8+ T cells (anti-CD8) and were co-labeled for platelets (anti-CD42b) and activated platelets (anti-CD62P, P-selectin). Data was collected by flow cytometry. Populations were gated based on CD4+ and CD8+. Percent of CD4+ cells with a platelet attached (A) and percent of CD8+ cells with a platelet attached (B) were calculated using FlowJo software. Populations were further gated based on PTCAs and the percent of activated platelets within CD4+ PTCAs (C) and CD8+ PTCAs (D) were calculated. Linear regression. N = 44–52. (DOCX) [file pone.0236966.s004.docx]

**
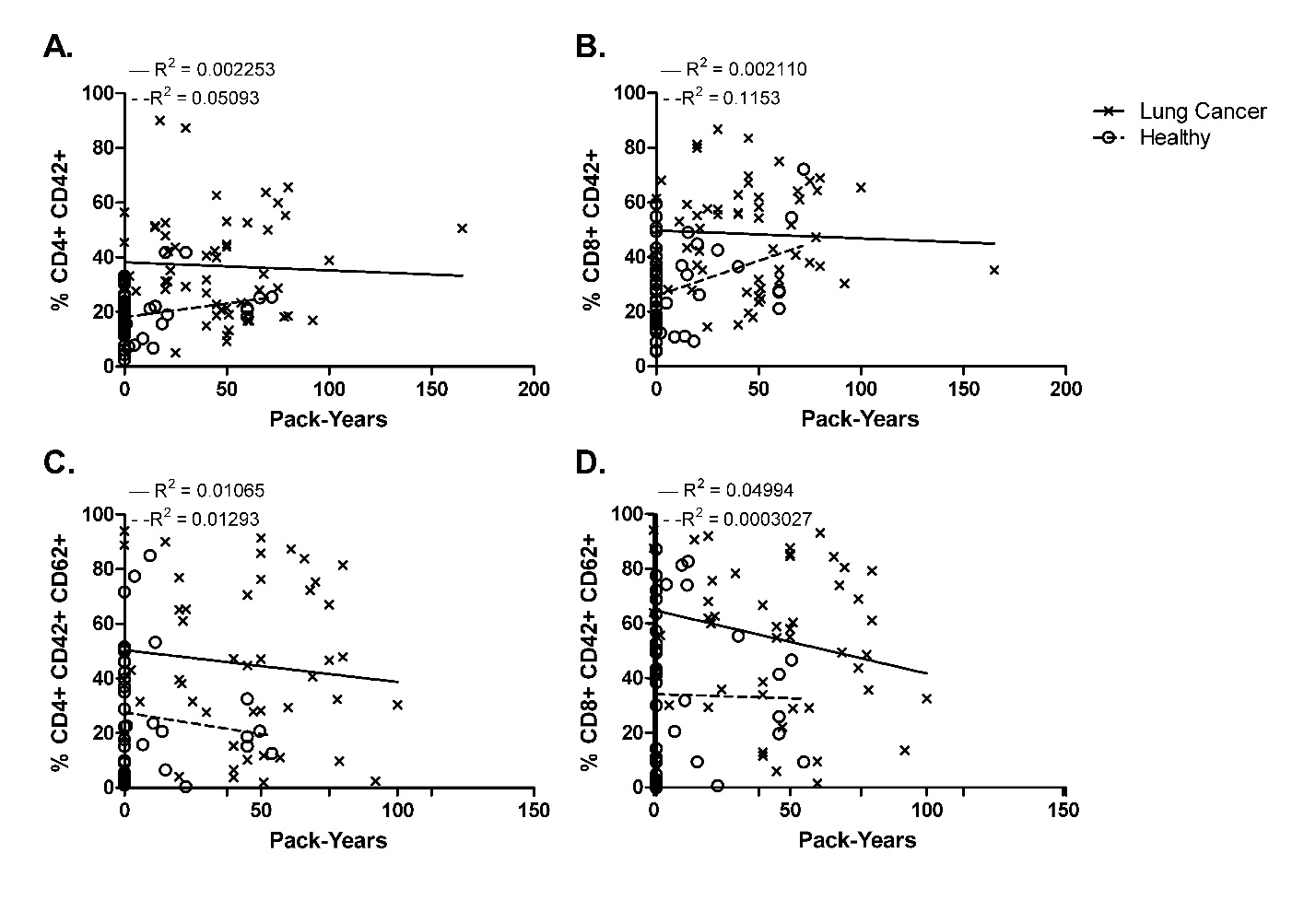
**

**Figure S3. PTCA formation as a variable of smoking history.** Pack-years were calculated as number of packs of cigarettes smoked per day multiplied by number of years participants smoked. Whole blood from healthy volunteers (○) or lung cancer patients ( x ) was labeled with markers for CD4+ T cells (anti-CD4) or CD8+ T cells (anti-CD8) and were co-labeled for platelets (anti-CD42b) and activated platelets (anti-CD62P, P-selectin). Data was collected by flow cytometry. Populations were gated based on CD4+ and CD8+. Percent of CD4+ cells with a platelet attached (A) and percent of CD8+ cells with a platelet attached (B) were calculated using FlowJo software. Populations were further gated based on PTCAs and the percent of activated platelets within CD4+ PTCAs (C) and CD8+ PTCAs (D) were calculated. Linear regression. N = 44-52.
