# Supplementary material for: Platelet-T cell aggregates in lung cancer patients: Implications for thrombosis
Source: PLoS One. 2020 Aug 10;15(8):e0236966. doi: 10.1371/journal.pone.0236966 (PMC7416940; doi:10.1371/journal.pone.0236966)
Supplement: S4 Fig — Whole blood from lung cancer patients and healthy volunteers was prepared as in Fig 6. Populations were gated based on CD4+ and CD8+. Percent of T cells with a platelet attached (A, B) and MFI of platelets (F, G) were calculated using FlowJo software. Populations were further gated based on PTCAs and the percent of free platelets (C) and MFI of free platelets (H) were determined. Percent of activated platelets (D, E) and MFI of activated platelets (I, J) within PTCAs were calculated. Optimal sensitivity and (1-specificity) were determined using GraphPad Prism and are plotted as gray lines. N = 44–52. (DOCX) [file pone.0236966.s005.docx]

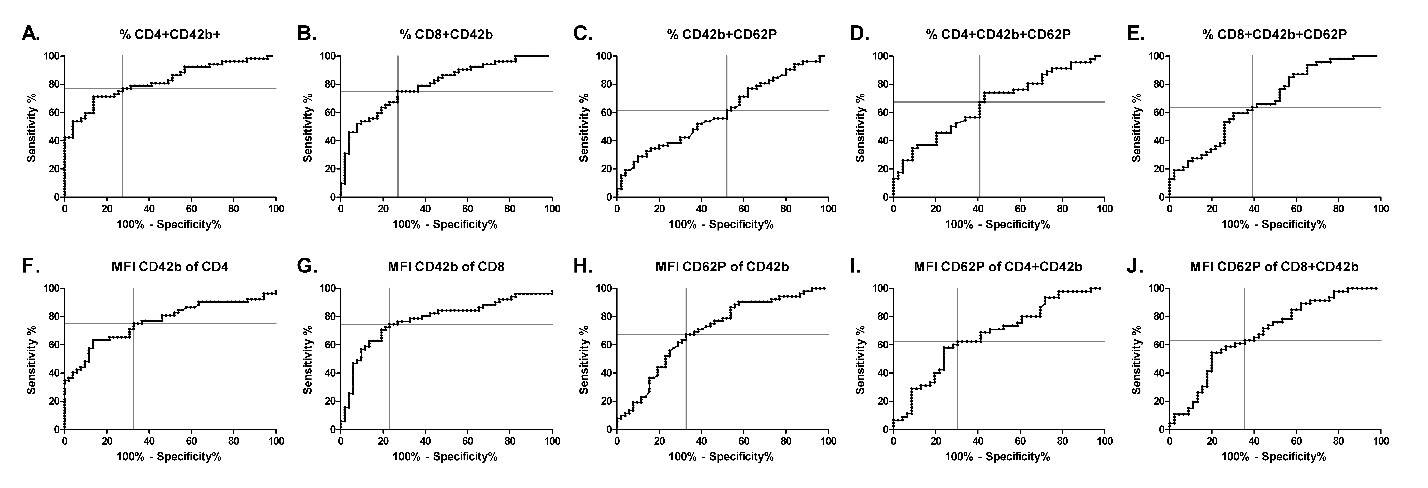


**Figure S4. ROC curves for lung cancer patients compared to healthy volunteers.** Whole blood from lung cancer patients and healthy volunteers was prepared as in Figure 6. Populations were gated based on CD4+ and CD8+. Percent of T cells with a platelet attached (A, B) and MFI of platelets (F, G) were calculated using FlowJo software. Populations were further gated based on PTCAs and the percent of free platelets (C) and MFI of free platelets (H) were determined. Percent of activated platelets (D, E) and MFI of activated platelets (I, J) within PTCAs were calculated. Optimal sensitivity and (1-specificity) were determined using GraphPad Prism and are plotted as gray lines. N = 44-52.
