# Supplementary material for: Platelet-T cell aggregates in lung cancer patients: Implications for thrombosis
Source: PLoS One. 2020 Aug 10;15(8):e0236966. doi: 10.1371/journal.pone.0236966 (PMC7416940; doi:10.1371/journal.pone.0236966)
Supplement: S5 Fig — Whole blood from lung cancer patients taking antiplatelet or anticoagulant medications (Meds) and those not taking any of these medications (No Meds) was prepared as in Fig 6. Populations were gated based on CD4+ and CD8+. Percent of T cells with a platelet attached (A) and MFI of platelets (C) were calculated using FlowJo software. Populations were further gated based on PTCAs and the percent of activated platelets (B) and MFI of activated platelets (D) within PTCAs were calculated. Asterisks above columns indicate a significant difference from the corresponding “CD42+CD62” (B) or “CD42” (D) free platelet population. Two-way ANOVA with Bonferroni post-test and Wilcoxon matched-pairs signed rank test. Error bars represent mean ± SD. N = 14–37. * p ≤ 0.05, ** p ≤ 0.01, *** p ≤ 0.001. (DOCX) [file pone.0236966.s006.docx]

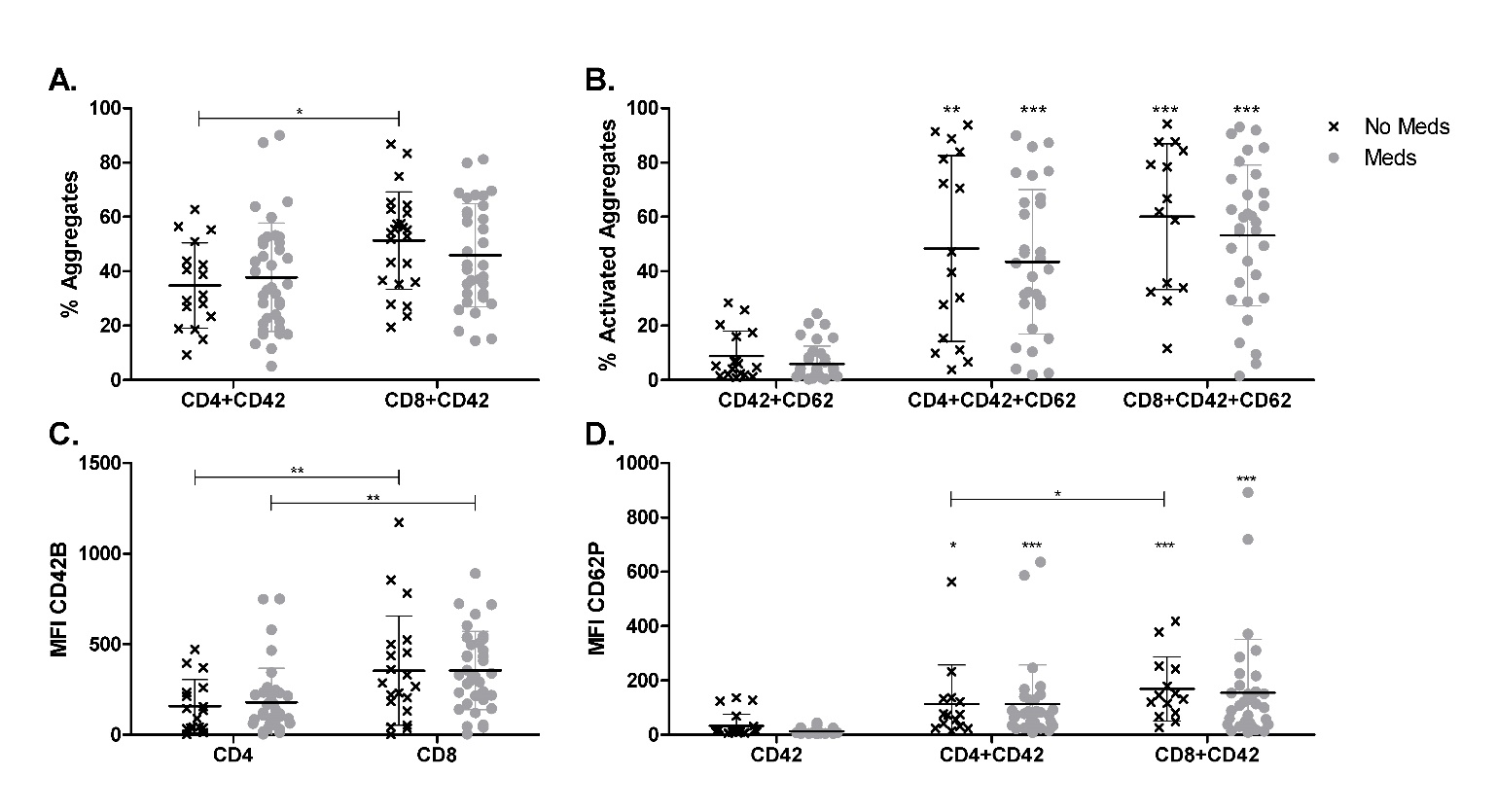


**Figure S5.** **Platelet-T cell aggregates in lung cancer patients by medication.** Whole blood from lung cancer patients taking antiplatelet or anticoagulant medications (Meds) and those not taking any of these medications (No Meds) was prepared as in Figure 6. Populations were gated based on CD4+ and CD8+. Percent of T cells with a platelet attached (A) and MFI of platelets (C) were calculated using FlowJo software. Populations were further gated based on PTCAs and the percent of activated platelets (B) and MFI of activated platelets (D) within PTCAs were calculated. Asterisks above columns indicate a significant difference from the corresponding “CD42+CD62” (B) or “CD42” (D) free platelet population. Two-way ANOVA with Bonferroni post-test and Wilcoxon matched-pairs signed rank test. Error bars represent mean ± SD. N = 14-37. * p ≤ 0.05, ** p ≤ 0.01, *** p ≤ 0.001.
